# Dynamic Monitoring of Circulating Tumor DNA to Predict the Risk of Non In Situ Recurrence of Postoperative Glioma: A Prospective Cohort Study

**DOI:** 10.1002/cam4.70733

**Published:** 2025-03-01

**Authors:** Guangzhong Guo, Ziyue Zhang, Jiubing Zhang, Dayang Wang, Sensen Xu, Shuang Wu, Kaiyuan Deng, Yage Bu, Zhiyuan Sheng, Jinliang Yu, Yushuai Gao, Zhaoyue Yan, Ruijiao Zhao, Meiyun Wang, Tianxiao Li, Xingyao Bu

**Affiliations:** ^1^ Department of Neurosurgery, Juha International Center for Neurosurgery Zhengzhou University People's Hospital Zhengzhou Henan China; ^2^ Department of Pathology Zhengzhou University People's Hospital, Henan Provincial People's Hospital, Henan University People's Hospital Zhengzhou Henan China; ^3^ Department of Radiology Zhengzhou University People's Hospital, Henan Provincial People's Hospital, Henan University People's Hospital Zhengzhou Henan China; ^4^ Henan Provincial Neurointerventional Engineering Research Center, Henan International Joint Laboratory of Cerebrovascular Disease, Henan Engineering Research Center of Cerebrovascular Intervention Innovation Zhengzhou Henan China; ^5^ Department of Cerebrovascular Disease Zhengzhou University People's Hospital, Henan Provincial People's Hospital, Henan University People's Hospital Zhengzhou Henan China

**Keywords:** biomarker, circulating tumor DNA, clonal evolution, glioma, risk stratification, tumor recurrence

## Abstract

**Background:**

Glioma recurrence can be divided into in situ recurrence and non‐in situ recurrence, and the mutation evolution of gliomas with different recurrence patterns is still unknown. We used sequential sequencing of circulating tumor DNA (ctDNA) to compare the somatic mutation profile and clonal evolution of gliomas with different recurrence patterns. To investigate the value of ctDNA in predicting early postoperative tumor recurrence and guiding prognosis stratification in patients with glioma.

**Methods:**

We prospectively recruited 92 patients with near‐total resection of gliomas from our center. Two hundred and thirty‐four postoperative tissue and Tumor In Situ Fluid (TISF) samples from 69 eligible patients were included in ctDNA analysis.

**Results:**

Among the 69 patients, 37 glioblastoma (GBM) patients experienced recurrence, and the median progression‐free survival (mPFS) was not significantly different between the situ recurrence group and the non‐in situ recurrence group (8.6 vs. 6.1 months). The ctDNA of recurrent tissue and TISF were significantly consistent. Before and after initial treatment, TISF‐ctDNA mutant allele fraction (MAF), subclonal mutation, and alterations in related pathways (lysine degradation and PI3K pathway) were negatively correlated with treatment response and PFS. Among recurrent GBM patients, EGFR mutations were the most common. Mutations related to the RTK‐RAS pathway (NF1) were most common in patients with situ recurrent GBM, while mutations in the MUC family and TP53 pathway (MUC16, CHEK2) were prevalent and continuously increased in patients with non‐in situ recurrent GBM.

**Conclusions:**

In glioma patients undergoing primary surgery, dynamic monitoring of ctDNA and genotyping can be used for early risk stratification, efficacy monitoring, and early recurrence detection, and provide a basis for clinical research to evaluate early therapeutic intervention.

## 1 Introduction

Glioma is the most common malignant primary brain tumor [1, 2]; its incidence increases with age. Despite aggressive first‐line therapy, recurrence is inevitable [3, 4, 5]. Studies have reported the evolutionary trajectory of glioblastoma at recurrence, suggesting that recurrent glioblastomas typically regrow from an oligoclonal origin, indicating minimal selective pressure for therapeutic measures [6]. The genomic pathways leading to recurrence are highly specific, generally classified into linear and divergent recurrence. The former exhibits extensive genetic similarity to the primary tumor, while the latter shares few genetic alterations and originates from cells branching early in tumorigenesis [7]. The TP53 pathway, TERT promoter mutations, and LTBP4 mutations in the TGF‐β pathway are potential predictors of glioblastoma recurrence [6, 7, 8]. However, these studies have used only tumor tissues, limiting longitudinal assessment of genetic variation. Comparative studies of genetic variation across different recurrence patterns are scarce. In this study, the recurrence of gliomas was divided into the local (in situ) recurrence group and the non‐in situ recurrence group, and the molecular evolution between the two groups was longitudinally studied to explore the molecular mechanism of different recurrence patterns of gliomas.

Circulating tumor DNA (ctDNA) is a promising biomarker to identify tumor mutation characteristics [9, 10, 11, 12], enabling real‐time tracking of tumor evolution and treatment response [13]. However, due to the limitations associated with glioma growth positions, cerebrospinal fluid (CSF) obtained by lumbar puncture is not an ideal source for evaluating glioma genetic characteristics [14, 15]. Previous studies from our group have shown that ctDNA from Tumor In Situ Fluid (TISF) can overcome these limitations, providing valuable information for diagnosis and prognosis [16, 17, 18, 19, 20]. TISF tumors are more representative than the DNA of cerebrospinal fluid, contributing to the sensitivity of tumor DNA and the clinical management of patients with glioma and clinical research. Tumor fluid in situ (TISF) collected through the postoperative tumor cavity is emerging as an attractive alternative.

In this prospective cohort study, we aimed to use longitudinal dynamic TISF‐ctDNA monitoring to evaluate the molecular and clonal evolution of gliomas and predict the recurrence risk of gliomas with different recurrence patterns. These results may assist in early recurrence prediction and intervention, ultimately improving patient management.

## 2 Materials and Methods

### 2.1 Study Design and Participants

This prospective study (ClinicalTrials.gov, NCT 05512325) was designed and conducted at Henan Provincial People's Hospital (Zhengzhou, China). From April 10, 2019 to October 16, 2023, 69 patients diagnosed with glioma (grade II, III,and IV) in Henan Provincial People's Hospital (HPPH) were included in this study. A total of 69 patients diagnosed with glioma (grade II, III, and IV) after primary surgery were selected from Henan Provincial People's Hospital (HPPH). Inclusion criteria were aged > 18 years old, near‐total resection of the lesion, and no history of malignancy in the last 5 years. The exclusion criteria are as follows: (a) having other infectious diseases or immunodeficiency diseases; (b) non‐neurological malignancies; (c) drug abuse; (d) serious mental illness; (e) uncontrolled diabetes. Sixty‐nine patients were eligible for analysis of postoperative ctDNA performance, and the baseline characteristics of the patients are shown in Table 1. This study has obtained HPPH (Zhengzhou, China) institutional review board and ethical committee approval. All patients and/or their legal representatives provided written informed consent to participate in the study and provide samples for tumor genetic profiling. All research procedures conformed to the principles of the Helsinki Declaration.

**TABLE 1:** Clinical characteristics of the study cohort at baseline (*N* = 69).

Characteristic	Patients (*n* = 69)
Age, median (range), y	53 (49–58)
<60 y	46 (66.7%)
Sex	
Male	32 (46.4%)
Histology	
Oligodendroglioma	12 (17.4%)
Astrocytome	13 (18.8%)
Glioblastoma	44 (63.8%)
First‐line therapy	
Chemotherapy	69 (100%)
Radiotherapy	23 (33.3%)
MGMT promoter methylation status	
Unmethylated	21 (30.4%)
Methylated	22 (31.9%)
Not reported	26 (37.7%)
ECOG performance status	
0	21 (30.4)
1	48 (69.6%)
Additional sequencing	
Tissue sequencing	43 (62.3%)
TISF sequencing	
Basel	69 (100%)
After treatment	67 (97.1%)
Recurrence	43 (62.3%)

### 2.2 Sample Collection, DNA Extraction, and Library Preparation

Tumor in Situ Fluid (TISF) samples were collected as previously described [17, 18, 19, 20, 21]. A small amount of TISF (0.5–2 mL) was obtained by syringe from the implanted reservoir sac every 4–8 weeks. TISF is the fluid present in the local surgical cavity. ctDNA profiles from tumor tissue and TISF samples can be used to assess the dynamic evolution of the tumor in real time, with 5 mL of blood collected as a germline DNA control.

Genomic DNA (gDNA) and cell‐free DNA (cfDNA) were extracted from fresh tissue, formalin‐fixed, paraffin‐embedded (FFPE) tissue, leukocytes, and tumor interstitial fluid (TIF) using kits (Kai Shuo, Thermo), respectively. Extraction procedures were performed according to the manufacturer's instructions. DNA was quantified using the Qubit dsDNA HS Assay Kit (Thermo) with a Qubit fluorescence quantifier, and its quality was assessed using the Agilent 4200 TapeStation (Agilent).

Commercial reagents and custom probes were used for library construction and hybridization capture. Briefly, Gdna (15 ng–200 ng) was cleaved into fragments ranging from 200 to 350 bp using a fragmenting enzyme. A self‐developed and customized index paired‐end adapter (SimcereDx) based on the Illumina platform was used. The cleaved DNA and cfDNA were subjected to end repair, A‐tailing, and adapter ligation with the use of a library preparation kit (Roche Diagnostics, Vazyme), respectively. Unattached adapters were removed using agcourt AMPure XP magnetic beads (Beckman Coulter). The ligation products were then subjected to PCR amplification to create a pre‐library for hybridization. The final library's quantification was conducted using the Qubit Fluorometer with the Qubit dsDNA HS Assay kit (Thermo Fisher), and the library's quality was assessed using the Agilent 4200 TapeStation (Agilent).

### 2.3 Library Sequencing and Bioinformatics Analysis

The qualified DNA libraries were sequenced using the Illumina NovaSeq6000 platform (Illumina, San Diego, CA) to generate 150 bp paired‐end reads. Adapter trimming and filtering of low‐quality bases were performed using the software fastp (v.2.20.0). The reads were then aligned to the reference genome (hg19, GRCh37 of UCSC) using the BWA‐MEM (v.0.7.17) algorithm. Duplicate reads in PCR were removed using Dedup and Error Correct. SNVs/indels were called and annotated using VarDict (v.1.5.7) and InterVar, respectively. These variants were then screened for common SNPs from public databases (1000 Genome Project, ExAC). CNVkit (dx1.1) was used to analyze CNVs, and factera (v1.4.4) was used to analyze fusion genes.

### 2.4 Treatment Regimens

#### 2.4.1 High Grade Glioma

Patients received concurrent chemoradiotherapy (TMZ, 75 mg/m^2^/d for 42 days) 4 weeks after surgery. Thereafter, TMZ was administered orally at 150 mg/m^2^/d every 4 weeks for 5 days, repeated every 28 days for 6 cycles.

Low grade glioma: Patients received postoperative chemotherapy (TMZ 150 mg/m^2^/d for 5 days) every 4 weeks for 6 cycles.

#### 2.4.2 Recurrent Glioma

Surgery was recommended. Patients who refused surgery were given bevacizumab (5 mg/kg, IV) combined with TMZ 150 mg/m^2^/d orally for 5 days, repeated every 21 days, for a total of 6 cycles.

#### 2.4.3 Bevacizumab‐Refractory Recurrent Glioma

Bevacizumab 3 mg/kg combined with sintilimab (an anti‐PD‐1 antibody)200 mg intravenously; This was repeated every 21 days for six cycles.

### 2.5 Analysis of the Consistency of Genetic Mutations

All samples were sequenced to obtain somatic mutations and driver gene variants, and ctDNA consistency analysis was performed after copy number alterations (CNAs) or nondriver gene variants were removed [22].

### 2.6 Mutation Clonality Analysis

If the mutated VAF at baseline exceeds 25% of the maximum VAF, the mutation is considered clonal, and if it is below this threshold, it is defined as subclonal. Newly acquired mutations in subsequent samples are often defined as subclonal mutations [23].

### 2.7 Statistical Analysis

The Kaplan–Meier curves for survival analysis. Using Cox proportional hazards models to determine each research variable associated with survival results. Continuous variables were compared using unpaired two‐tailed *t*‐tests, and categorical variables between groups were compared using the chi‐square test or Fisher's exact test. Overall survival (OS) was defined as the time from the initiation of first‐line therapy to the death or last follow‐up time. Progression‐free survival (PFS) was defined as the time from the initiation of first‐line therapy to first disease progression or death. The treatment response was evaluated according to the Neuro‐oncology Response Evaluation (RANO) criteria. All statistical analyses were performed using R software (version 3.5.3). *p* < 0.05 was considered to be statistically Significant. The study funders had no role in study design, data collection, analysis, interpretation, or manuscript writing.

## 3 Results

### 3.1 Characteristics of the Study Cohort

**FIGURE 1:**
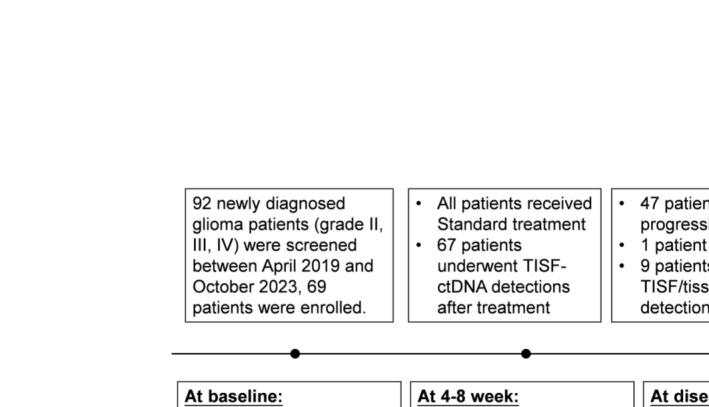
Flowchart of the study design and participants. The study design and sample collection timepoints are shown with the number of patients and available samples. PD, progressive disease; TISF, tumor in situ fluid.

The flowchart of the study is shown in Figure 1. Sixty‐nine patients were included in the postoperative ctDNA performance analysis. All patients received first‐line treatment after tumor resection. Administration was not interrupted during any treatment cycle. The median age of all patients was 53 years (49–58 years), and 32 (46.4%) were male. According to the clinicopathological examination, 44 cases (63.8%) were GBM, 13 cases (18.8%) were astrocytoma, and 12 cases (17.4%) were oligodendroglioma. The median PFS and OS were 14.9 months (95% CI: 9.0–20.8) and 30.4 months (95% CI: 24.1–36.8), respectively. The median progression‐free survival (mPFS) was not significantly different between the situ recurrence group and the non‐in situ recurrence group (8.6 vs. 6.1 months).

TISF samples were obtained from all patients at baseline, and plasma samples were collected to exclude germline mutations. Forty‐five patients had paired primary tumor tissue at baseline. TISF samples were collected from 67 patients 4–8 weeks after first‐line treatment. By the data deadline (October 2023), 47 patients were diagnosed with disease progression, and 33 cases of death. The median time to disease progression was 9.9 months (95% CI: 5.6–14.3) in GBM patients, while the median time to disease progression was not reached in oligodendroglioma and astrocytoma patients.

### 3.2 Concordance of Genomic Alteration Detection in TISF and Tumor Tissue Samples

**FIGURE 2:**
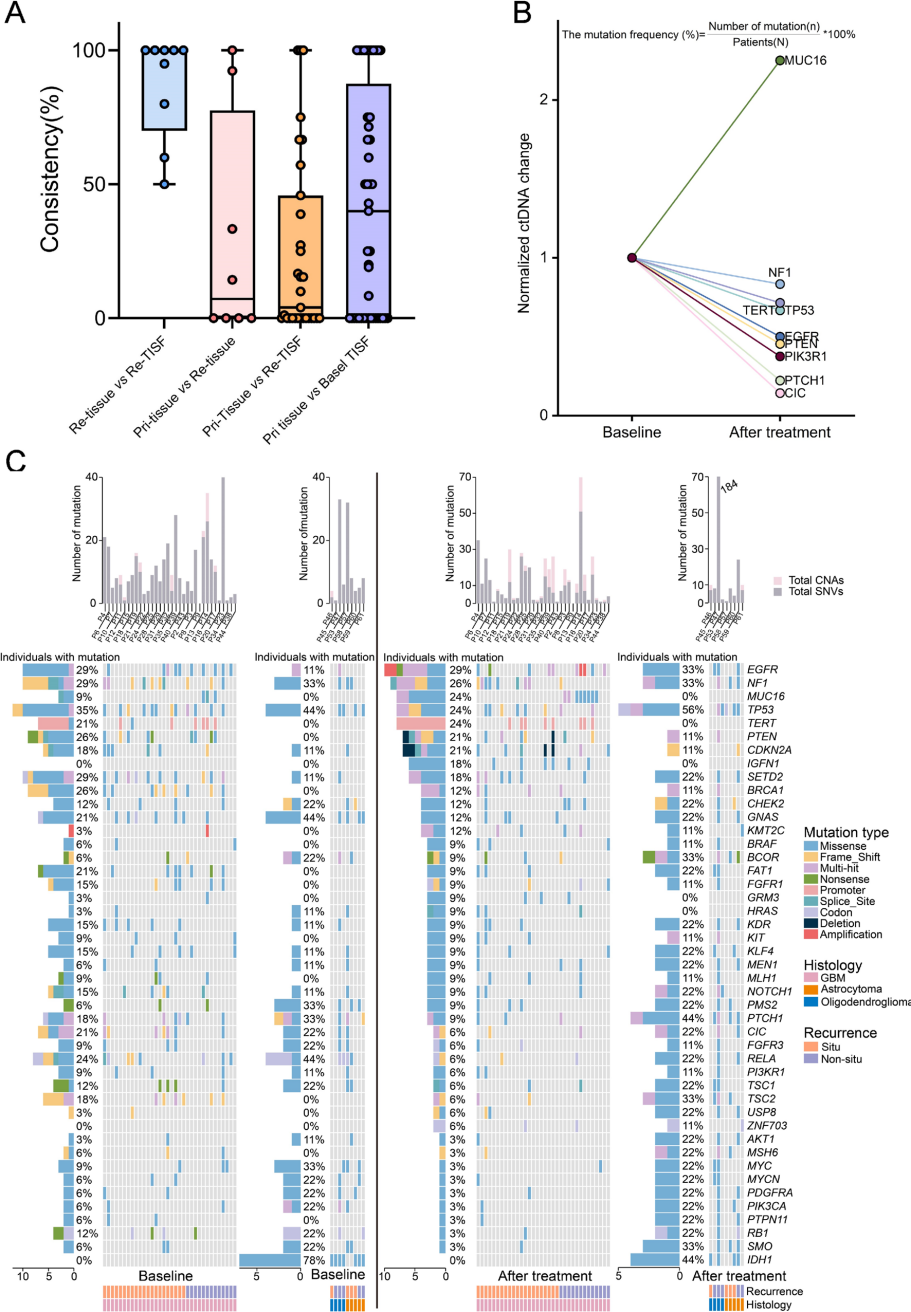
Concordance analysis and baseline to relapse genomic profiling. (A) Correlation between tissue and TISF. *P*‐values were calculated using an unpaired two‐tailed *t*‐test. (B) Spider plot of baseline and post‐treatment changes in gene mutation rates in 42 GBM patients. Staining was performed according to gene, and the gene mutation rate after treatment was divided by the baseline gene mutation rate. The mutation frequency (%) = Number of mutation(n)/Patients(N)*100%. (C) Mutational landscape of 44 glioma patients from baseline to recurrence, showing a number of somatic mutations in each patient (top), the mutation frequency of each gene (left).

Tissue and TISF samples from 45 glioma patients were available for concordance analysis. Among them, the patient diagnosed with pseudoprogression after surgery (Patient 37) had no detectable genomic alterations in either TISF or recurrent tissue before recurrence. A median of 40.0% (95% CI: 0%–60.0%), 4.0% (95% CI: 0%–27.3%), and 7.1% (95% CI: 0%–92.3%) genomic alterations were detected in the ctDNA of primary tissues in basal‐TISF, relapse‐TISF, and relapse tissues, respectively. There was low consistency between recurrent tumor ctDNA and primary tumor ctDNA (Figure 2A). In contrast, genomic alterations were detected in a median of 100% (95% CI: 60.0%–100%) of relapsed tissue in relapsed TISF‐ctDNA (Figure 2A). We further demonstrate the concordance between TISF and tissue ctDNA and that TISF can characterize gene evolution in gliomas in real time.

### 3.3 ctDNA Displays Unique Genetic Profiles of Glioma Recurrence

Comparing the common gene profiles of 69 patients with different grades of glioma at different time points (Figure S1), TP53 mutations were the most commonly altered genes in GBM, followed by NF1 mutations. After comparing common genetic alterations at baseline and after treatment in 42 patients with glioblastoma, we found that only MUC16 gene mutation frequency increased, while the remaining genes decreased (Figure 2B).

**FIGURE 3:**
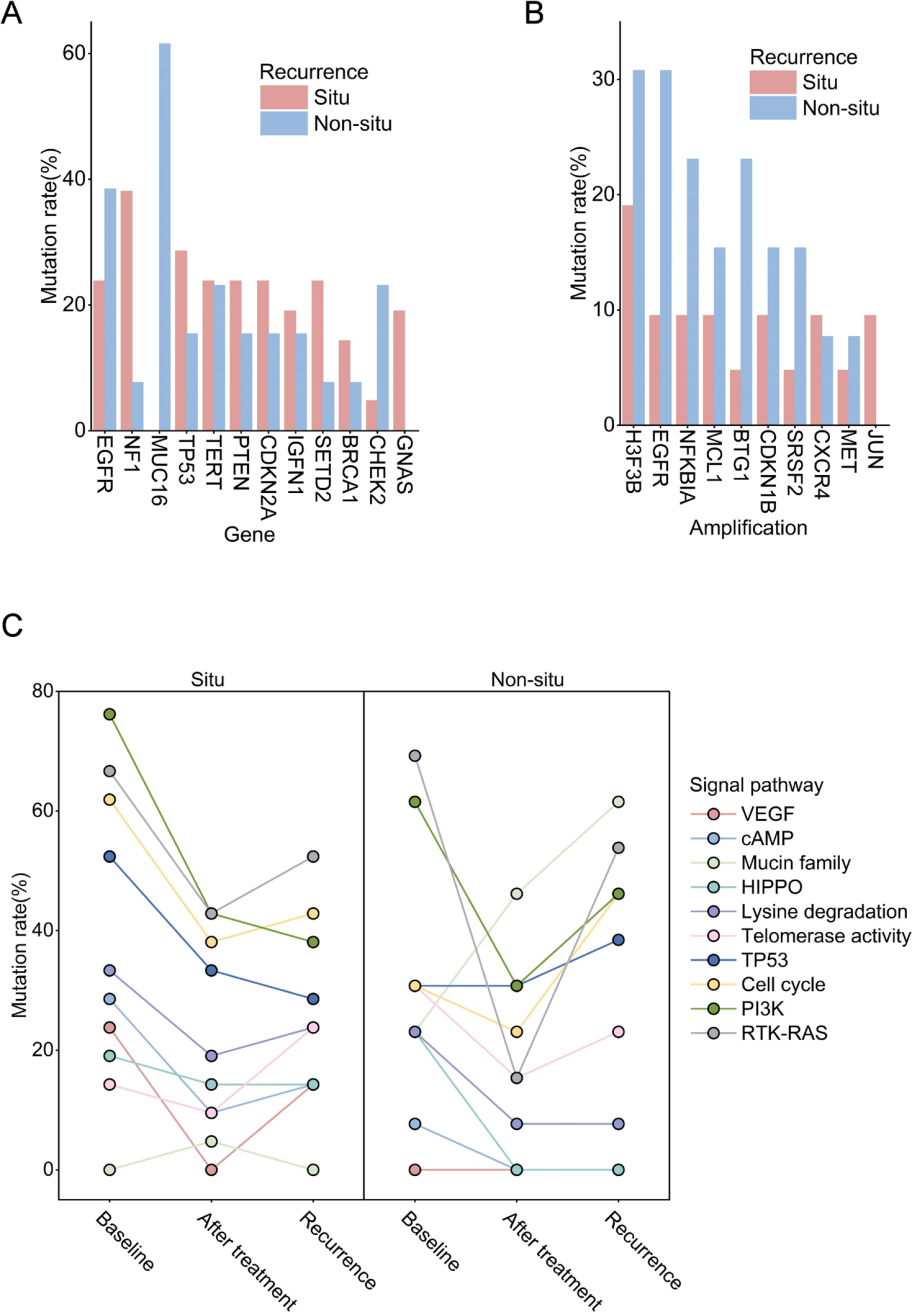
Genetic changes in glioblastoma patients with different recurrence patterns. Mutation rates of genes (A) and copy number variations (B) in patients with recurrent glioblastomas at different patterns of recurrence. (C) Changes of signaling pathways from surgery to recurrence in glioblastoma patients with different recurrence patterns.

44/47 patients with recurrent TISF ctDNA samples, including GBM (*n* = 35), oligodendroglioma (*n* = 4), and astrocytoma (*n* = 5), including 25 cases of in situ recurrence, 18 cases of non‐in situ recurrence, and one case (P37) of pseudoprogression; EGFR is the most common mutated gene in rGBM, followed by NF1 (Figure 2C). In rGBM, MUC16 and CHEK2 mutation rates were higher in the non‐in situ recurrence subgroup than in the in situ recurrence subgroup, whereas NF1 was more common in the in situ recurrence subgroup (Figure 3A).

The detection rate of CNAs in patients with non‐in situ recurrence (61.5%, 8/13) was higher than that in patients with in situ recurrence (47.6%, 10/21). H3F3B amplification was the most common CNA. The mutation rates of common CNAs were higher in the non‐in situ recurrence group than in the in situ recurrence group (Figure 3B).

Finally, the alterations of signaling pathways in different recurrence patterns were counted. Somatic mutations were classified according to oncogenic signaling pathways previously reported in TCGA and KEGG (Kyoto Encyclopedia of Genes and Genomes). We found that the signaling pathway changes differently in gliomas with different recurrence patterns. Mutations associated with the RTK‐RAS pathway were the most common in patients with in situ rGBM, and we observed a continuous decline in TP53 and PI3K pathway‐related mutations. In contrast, mutations associated with the MUC family pathway were most common and consistently increased in patients with non‐in situ rGBM (Figure 3C).

### 3.4 Clonal and Subclonal Evolution During First‐Line Treatment of Gliomas

**FIGURE 4:**
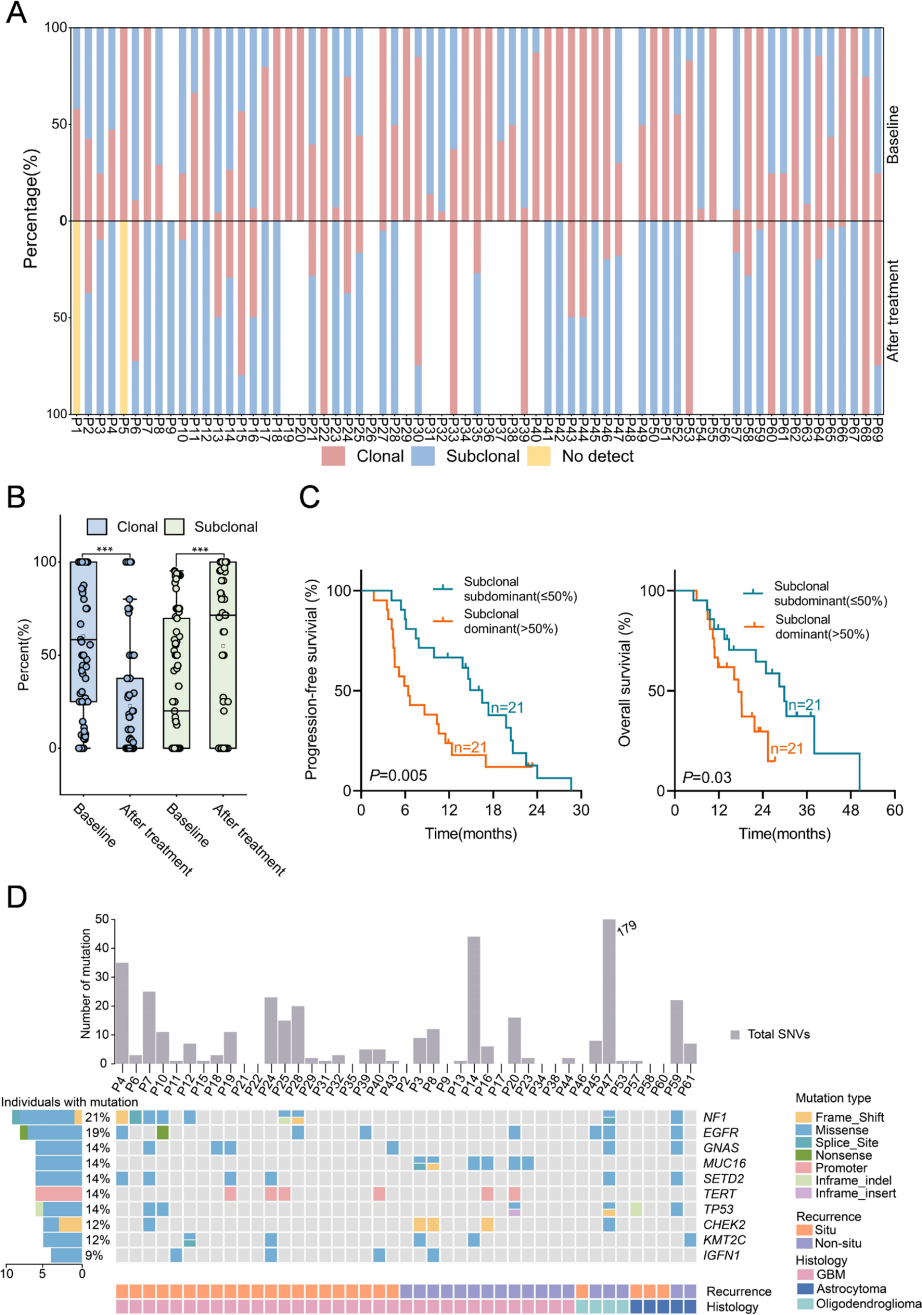
Evolution of clonal and subclonal mutations from baseline to relapse. (A) Percentage of clonal and subclonal mutations at baseline and 4 weeks after treatment. (B) The proportion of clonal and subclonal mutations in TISF samples at baseline and after 4 weeks of treatment. (C) Kaplan–Meier curves of progression‐free survival and overall survival according to subclonal mutations > 50% at 4 weeks after treatment. (D) Subclonal gene changes in gliomas with different recurrence patterns.

We defined clonal and subclonal mutations as described in the Methods section. At baseline, four TISF samples were negative for ctDNA. At least one subclonal mutation was identified in 56.5% (39/69) of TISF samples (Figure 4A). TP53 was enriched as a clonal mutation in TISF samples from rGBM patients. The overall proportion of clonal mutations in TISF samples decreased after treatment, mainly due to the clearance of clonal mutations and the acquisition of new subclonal mutations (Figure 4A,B). Among the mutations detected in TISF samples collected after treatment, subclonal mutations > 50% of GBM patients have a worse prognosis (PFS: 6.4 months vs. 16.5 months, HR = 2.578, *p* = 0.005; OS: 17.3 months vs. 29.8 months, HR = 1.721, *p* = 0.03; Figure 4C).

We then analyzed the evolution of clonal and subclonal mutations in patients with different recurrence patterns. The common subclonal mutation in the in situ recurrence group was NF1, while the common subclonal mutation in the non‐in situ recurrence group was MUC16 (Figure 4D).

### 3.5 Independent Predictive Value of ctDNA

**FIGURE 5:**
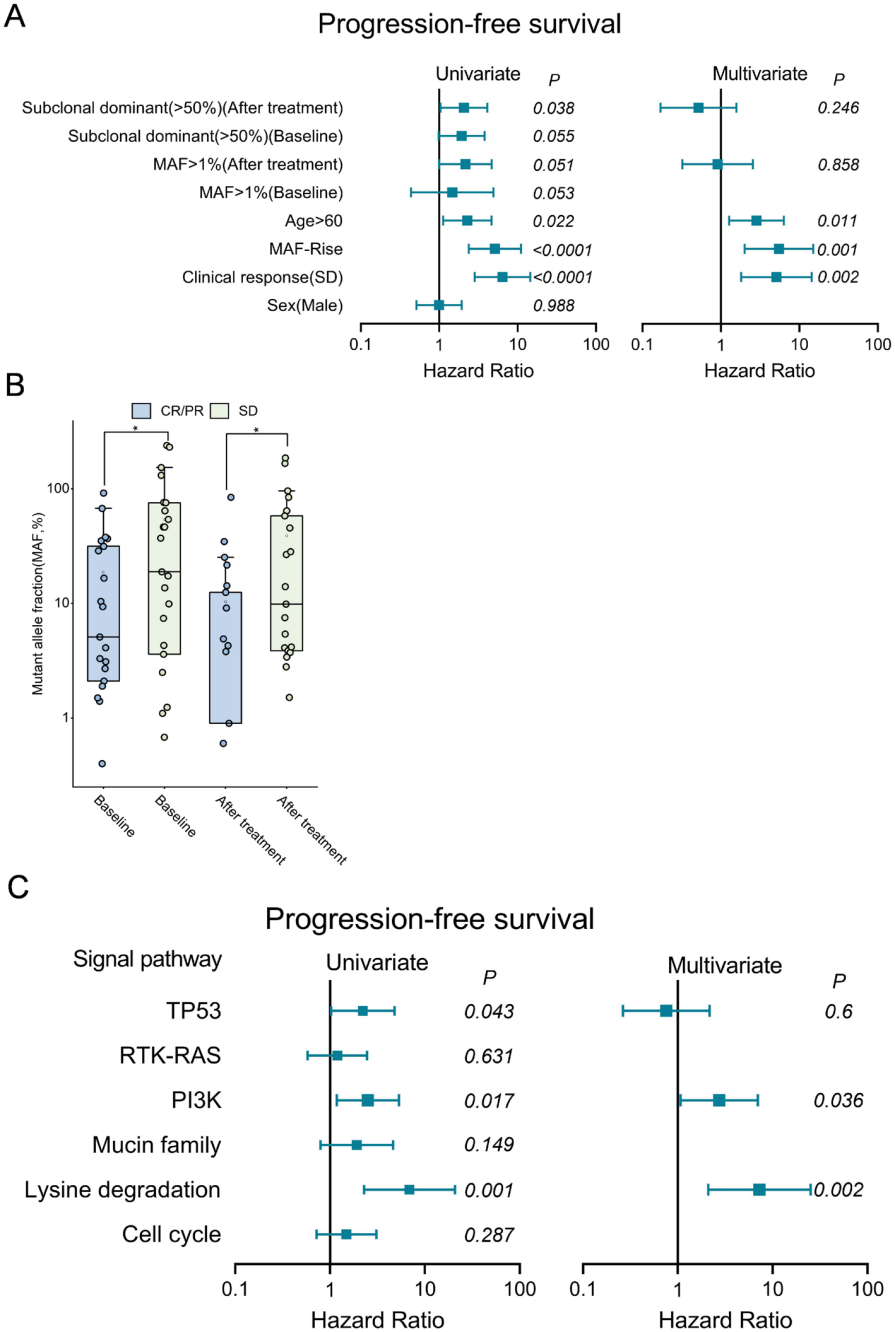
Independent predictive value of ctDNA. (A) Univariate and multivariate analysis of glioblastoma recurrence prediction with multiple clinical variables and longitudinal ctDNA. (B) The distribution of MAF of different imaging responses at different times. *P*‐values were calculated using an unpaired two‐tailed *t*‐test. (C) Univariate and multivariate COX analysis of signaling pathway alterations to predict glioblastoma recurrence.

Of the 69 patients enrolled in the trial, all had biopsy samples available for ctDNA analysis, and 36 patients (52.2%) had TISF data available for TMB assessment. All patients were assessed for response to first‐line therapy according to RANO criteria; 21 patients (30.4%) achieved CR, 22 patients (31.9%) achieved PR, and 26 patients (37.7%) achieved SD. To explore the prognostic value of ctDNA levels for each TISF sample at different time points, we calculated the MAF of all mutations detectable for each sample. By assessing the prognostic value of different MAF thresholds, 1%MAF was selected. Univariate Cox regression analysis showed that age (> 60 years), imaging evaluation (SD vs. CR/PR), MAF after treatment > 1%, MAF change (Rise vs. Down), and subclonal dominance after treatment (> 50%) were significantly associated with PFS in GBM patients. In multivariate analysis, age, imaging evaluation (SD vs. CR/PR), and MAF change were independent risk factors (Figure 5A).

The ctDNA level at baseline and after treatment was associated with a significant response to treatment. SD patients had significantly higher levels than CR/PR patients (unpaired two‐tailed *t*‐test, *p* = 0.032, *p* = 0.034; Figure 5B). We then calculated whether the changes in signaling pathways could predict prognosis. We found that gene alterations related to lysine degradation, TP53 and PI3K pathway after treatment were significantly associated with PFS in rGBM, and lysine degradation and PI3K pathway were independent risk factors (Figure 5C).

## 4 Discussion

This study demonstrates, for the first time, that dynamic changes in TISF‐ctDNA in gliomas can be used to monitor mutation evolution during treatment, suggesting that continuous ctDNA monitoring may provide an early and reliable predictor of therapeutic response and clinical benefit in glioma. By examining the dynamic changes in TISF‐ctDNA, the study reveals its potential in predicting treatment response and monitoring mutations, thereby contributing to our understanding of its clinical utility in glioma therapy.

Liquid biopsies offer the advantage of enabling real‐time monitoring of tumor evolution and predicting treatment response [24]. Initial evidence indicates that the most significant selection pressures may arise early in the development of glioblastoma, emphasizing the need for detailed molecular analysis at the outset to achieve optimal management [12, 25, 26]. Studies have reported an association between ctDNA found in plasma and CSF and tumor response [27, 28, 29, 30]. However, the presence of the blood–brain barrier prevents ctDNA from being released from intracranial tumors into the peripheral plasma [31, 32]. In addition, due to the location of glioma growth, the positive rate and representativeness of ctDNA in cerebrospinal fluid samples were affected, and the data of two related studies were 49.4% and 63.7%, respectively [14, 15, 22, 29]. Our previous studies have demonstrated the feasibility of TISF as a stable source of glioma liquid biopsy and that TISF is a more sensitive source of glioma DNA than cerebrospinal fluid, providing new insights for precision medicine in brain tumors [16, 17, 18, 19, 20]. More importantly, we found that ctDNA positivity was prevalent in baseline TISF samples, and in COX proportional hazards analysis, MAF > 1% in baseline TISF samples was not a poor prognostic factor for glioma patients (HR:1.46, *p* = 0.533), which was inconsistent with previous studies. Previous studies have reported that positive ctDNA in baseline CSF samples is associated with a higher disease burden and poor prognosis in glioma patients [29]. Although our research group found that ctDNA positivity was prevalent in baseline TISF samples, in COX proportional risk analysis, MAF > 1% in baseline TISF samples was not a factor for poor prognosis of glioma patients (HR: 1.46, *p* = 0.533), which was inconsistent with the results of previous studies. This inconsistency may be because positive CSF is related to tumor load and distance from the nearest ventricle, and the prognosis is worse when ctDNA is positive in CSF early after surgery.

We found that TP53 was consistently enriched as clonal mutations throughout the process. One study showed that the majority (90.5%) of TP53 and PIK3CA/PIK3R1 mutations were clonal and may play a founder role in GBM [7]. At the same time, the increased proportion of subclonal mutations in GBM containing alterations in the TP53 pathway may indicate an improved tolerance to DNA damage or apoptosis inhibition [33, 34, 35]. Their data also suggest that an increased frequency of subclonal mutations is associated with relatively favorable event‐free survival. The authors suggest that further research is necessary and provide two explanations for the paradox. Firstly, they propose that the longer interval between tumorigenesis may signify a slower disease progression, thereby allowing more time for subclones to proliferate to detectable levels. Alternatively, they propose that the absence of dominant invasive clones may indicate a reduced tumor growth rate attributed to a higher number of cells competing for cranial space. However, our study showed that an increase in the proportion of subclonal mutations was associated with a poor prognosis, and the accumulation of subclonal mutations was observed throughout the process.

Tumor recurrence is the main cause of death in glioma patients. It is difficult to perform biopsies again at the time of recurrence, and the lack of data on tumor characteristics may prevent clinicians from selecting effective second‐line treatment strategies. Recent studies by the Glioma Longitudinal Analysis (GLASS) Consortium report time‐series ctDNA sequencing of a large number of primary and recurrent glioma pairs and establish the evolutionary molecular profile of adult diffuse gliomas [25]. However, the research on in situ recurrence and non‐in situ recurrence of glioma still needs to be clarified. This study demonstrated that TISF‐ctDNA could characterize molecular signatures at recurrence, revealing intratumor heterogeneity under different recurrence patterns. We found that different recurrence patterns of gliomas had different driver mutations, and genetic alterations of some genes were enriched in different gliomas, among which MUC16 was enriched in patients with non‐in situ recurrence. The MUC gene family encodes mucin, a high molecular weight glycoprotein [36]. There are 21 MUC genes in the human genome encoding secretory and membrane mucin [37]. Evidence shows that the MUC protein regulates tumor cell proliferation, growth, apoptosis, and chemical tolerance [38, 39]. Pan‐cancer analyses involving somatic mutations in 10,195 samples and mRNA expression profiles in 9850 samples from 30 solid tumors found that immune cells were more abundant in the MUC16 mutant tumor microenvironment, and MUC16 mutations were associated with immune response‐related factors associated with checkpoint inhibitor therapy [40]. Compared with MUC16 wild‐type tumors, tumors with MUC16 mutations showed a higher tumor mutation load and more abundant neoantigens, indicating increased tumor immunogenicity. MUC16 plays a dual role in tumor immunity. On the one hand, MUC16 can inhibit tumor immunity by binding to inhibitory receptors on the surface of immune cells such as natural killer (NK) cells and macrophages [41, 42, 43]. On the other hand, MUC16 promotes the maturation of dendritic cells (DC). Neoantigens generated by MUC16 mutations can activate CD8 + T cells and enrich various tumor‐infiltrating immune cells to promote tumor immunity [40, 44, 45]. We have identified a high frequency of MUC16 mutations in patients with non‐in situ relapsed glioma and have begun anti‐PD‐1/PD‐L1 therapy trials in these patients. Dynamic monitoring of TISF‐ctDNA analysis from postoperative to recurrence will help identify changes in gene therapy targets during glioma progression, enabling treatment strategies to adapt to the molecular characteristics of the tumor at each time point. This research can reduce the long‐term secondary effects of unnecessary treatments and thus select the optimal personalized treatment [6, 7, 8, 46]. TISF samples can be used as part of the standard of routine postoperative care for glioma. With the implementation of TISF‐ctDNA testing in clinical practice, valuable information can be obtained from each patient, so that glioma patients can further benefit from precision medicine.

We also explored how ctDNA predicts response after treatment initiation. The ability to predict early whether a patient will benefit from treatment allows patients who have a poor response to treatment to switch to more effective treatment earlier, thereby increasing the chance of benefit and limiting unnecessary exposure. While improvements in survival were observed at 4 weeks of treatment when ctDNA was reduced, some patients survived longer despite an increase in ctDNA at 4 weeks. One possible explanation may be due to the dynamics of ctDNA release during treatment [47, 48, 49, 50]. ctDNA levels may increase dramatically after the initiation of therapy due to tumor cell death, resulting in the release of tumor DNA, which decreases as the tumor burden decreases. Therefore, we assessed the further changes in ctDNA levels observed beyond 8 weeks in patients with more prolonged survival, and we found a decrease in ctDNA levels. These data suggest that further optimization of the timing of ctDNA assessment after treatment initiation will be critical [22].

## 5 Conclusion

We prospectively recruited a cohort for dynamic ctDNA monitoring of gliomas after surgery and found that postoperative ctDNA is a promising biomarker for risk stratification and recurrence prediction of gliomas. In addition, we observed that a number of genetic mutations and associated pathways were associated with relapse patterns in early glioblastoma, and an increased accumulation of subclones was observed. In summary, sequence ctDNA analysis combined with tumor genome sequencing can provide accurate information for predicting and monitoring tumor recurrence, help to understand the evolution and mechanism of different recurrence patterns, and optimize individualized multimodal treatment strategies.

## Author Contributions

**Guangzhong Guo:** conceptualization (lead), data curation (equal), formal analysis (lead), investigation (lead), methodology (lead), project administration (equal), resources (equal), software (equal), supervision (equal), validation (lead), visualization (lead), writing – original draft (lead), writing – review and editing (equal). **Ziyue Zhang:** data curation (equal), project administration (equal). **Jiubing Zhang:** data curation (equal), project administration (equal). **Dayang Wang:** data curation (equal), project administration (equal). **Sensen Xu:** data curation (equal), project administration (equal). **Shuang Wu:** data curation (equal). **Kaiyuan Deng:** data curation (equal). **Yage Bu:** data curation (equal). **Zhiyuan Sheng:** data curation (equal). **Jinliang Yu:** data curation (equal). **Yushuai Gao:** data curation (equal). **Zhaoyue Yan:** data curation (equal). **Ruijiao Zhao:** supervision (equal). **Meiyun Wang:** supervision (equal). **Tianxiao Li:** supervision (equal). **Xingyao Bu:** conceptualization (equal), funding acquisition (lead), project administration (lead), resources (lead), supervision (lead), writing – review and editing (lead).

## Conflicts of Interest

The authors declare no conflicts of interest.

## Supporting information


Figure S1.

## Data Availability

The datasets used or analyzed during the current study are available from the corresponding author upon reasonable request.
