# Supplementary material for: Dynamic Monitoring of Circulating Tumor DNA to Predict the Risk of Non In Situ Recurrence of Postoperative Glioma: A Prospective Cohort Study
Source: Cancer Med. 2025 Mar 1;14(5):e70733. doi: 10.1002/cam4.70733 (PMC11871513; doi:10.1002/cam4.70733)
Supplement: Supplementary file 1 — Figure S1. [file CAM4-14-e70733-s001.pdf]

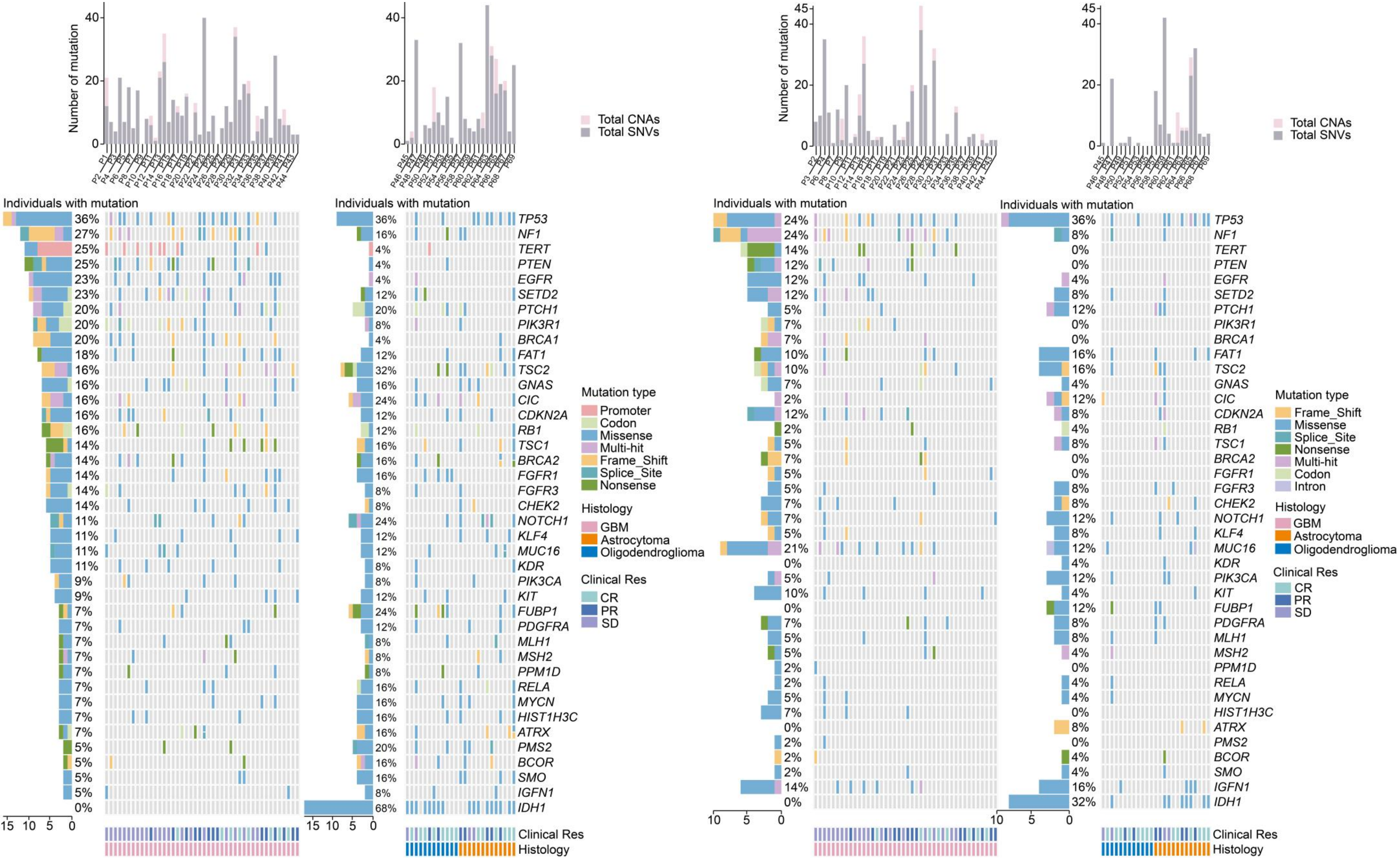

Figure S1. Mutational landscape of 69 glioma patients at baseline (left) and after initial treatment (right), showing the number of single nucleotide variants (SNVs) and copy number alterations (CNAs, top) for each patient, and the mutation frequency for each gene (left).
